# Molecular genetic and clinical evaluation of three Chinese families with X-linked ocular albinism

**DOI:** 10.1038/srep33713

**Published:** 2017-02-17

**Authors:** Xuan Zou, Hui Li, Lizhu Yang, Zixi Sun, Zhisheng Yuan, Huajin Li, Ruifang Sui

**Affiliations:** 1 Department of Ophthalmology, Peking Union Medical College Hospital, Peking Union Medical College, Chinese Academy of Medical Sciences, Beijing, China

## Abstract

X-linked ocular albinism (OA1) is an X-linked inherited disease characterized by hypopigmentation of the fundus and nystagmus. Our study performed mutation analysis of the G protein-coupled receptor 143 gene (*GPR143*) and assessed the clinical characteristics of OA1 in three Chinese families. Three novel mutations, c.333_360+14del42insCTT, c.276G>A (p.W92X), and c.793C>T (p.R265X), were identified in *GPR143* by PCR followed by Sanger sequencing in these families. All affected individuals presented with nystagmus, photophobia, poor visual acuity, foveal hypoplasia and varying degrees of hypopigmentation of the fundus. The fundus of female carriers showed pigmented streaks alternating with hypopigmented streaks. These results allowed us to expand the spectrum of mutations in *GPR143* and phenotypes associated with ocular albinism.

Albinism consists of a rare group of clinical and genetic abnormalities caused by defects in melanin synthesis involving the hair, skin, and eyes. There are two main subtypes of albinism: oculocutaneous albinism and ocular albinism. The birth prevalence of X-linked ocular albinism (OA1; MIM 300500) is approximately 1 in 50,000. OA1 occurs almost exclusively in males, and results in only ocular abnormalities, including impaired visual acuity, nystagmus, photophobia, foveal hypoplasia, hypopigmentation of iris and fundus^1^. The fundus of female asymptomatic carriers exhibits a mottled pattern of pigmentation, which is special characteristic for OA1.

OA1 is caused by mutations in the G protein-coupled receptor 143 gene (*GPR143*) (OMIM 300808), originally called *OA1*, which is located at Xp22.32^2^. *GPR143* encodes a protein that binds to heterotrimeric G proteins and is highly expressed in melanocytes and the retinal pigment epithelium (RPE)^2^. It encodes a 404 amino acid protein predicted to be a membrane protein essential for development and maturation of melanosomes^3^. Unlike other GPCRs, *GPR143* is not a transmembrane protein on the cell surface, but is exclusively localized to intracellular organelles, namely lysosomes and melanosomes^4^. It has been postulated that ocular albinism is a disorder of melanin secretion from the melanosome into keratocytes rather than a deficit in melanin synthesis. Mutant protein may lead to failure of melanosomes to bud off the endoplasmic reticulum. Instead, melanosomes are likely to aggregate as melanin accumulates, leading to the formation of giant melanosomes^5^,^6^.

Various types of mutations in *GPR143* have been identified in patients from different countries; however, the characteristics of OA1 have not been well defined in Asians. X-linked OA1 in the Chinese population has been rarely reported^7^,^8^,^9^,^10^,^11^,^12^,^13^,^14^,^15^. As iris and fundus hypopigmentation is not obvious among the Chinese patients, it is difficult to distinguish OA1 from other congenital eye diseases, such as Leber congenital amaurosis (LCA), achromatopsia or congenital motor nystagmus (CMN). Children are always uncooperative for detailed retinal structure and function evaluation. In addition, Lack of awareness of the clinical features of ocular albinism by health care providers leads to frequent misdiagnosis. In this study, we describe the gene mutation and clinical manifestations of OA1 patients and carriers from three unrelated Chinese families. Our data expands the spectrum of phenotypes and mutations in the *GPR143* gene in this population.

## Methods

### Recruitment of subjects

All participants were identified at the Ophthalmic Genetics Clinic at Peking Union Medical College Hospital (PUMCH), Beijing, China. Written informed consent was obtained either from the participating individuals or their guardians. This study was approved by the Institutional Review Board of PUMCH and adhered to the tenets of the Declaration of Helsinki and the Guidance on Sample Collection of Human Genetic Diseases by the Ministry of Public Health of China.

### Clinical evaluations

A full medical and family history was taken. Patients underwent a detailed ophthalmic examination, including best-corrected visual acuity (BCVA) according to decimal E charts, slit-lamp biomicroscopy, dilated indirect ophthalmoscopy and fundus photography. The retinal structure was examined by optical coherence tomography (OCT, Topcon, Tokyo, Japan and Optovue Inc., Fremont, CA) and fundus autofluorescence (FAF, Heidelberg Engineering, Heidelberg, Germany). Electrophysiological assessment (Roland Consult, Wiesbaden, Germany) included a full-field electroretinogram (ERG) to test the entire retina and multi-channel visual evoked potential (VEP) to identify possible abnormal optic nerve decussation. The test protocols conformed to the standards of the International Society for Clinical Electrophysiology of Vision (www.iscev.org).

### Genetic studies

Genomic DNA was isolated from peripheral leukocytes using the QIAamp DNA Blood Midi Kit (Qiagen, Hilden, Germany) according to the manufacturer’s protocol. *GPR143* exons and adjacent sequences were amplified by the polymerase chain reaction (PCR) using primers published previously^7^. After purification, amplicons were sequenced using forward and reverse primers on an ABI 3730 Genetic Analyzer (ABI, Foster City, CA). Sequences were assembled and analyzed with Lasergene SeqMan software (DNASTAR, Madison, WI). The results were compared with the *GPR143* reference sequence (NM_000273.2). All available family members were Sanger sequenced in order to confirm the segregation of mutations.

## Results

### Clinical phenotype

**Figure 1:**
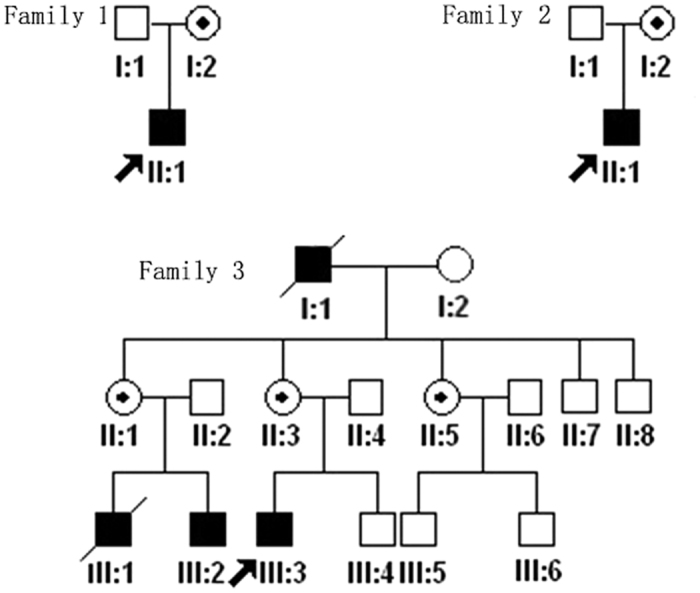
Pedigrees of the three families. Black filled symbols indicate patients affected with OA1 in each family. Dot-marked symbols represent carriers. The proband is marked by an arrow in each family.

**Figure 2:**
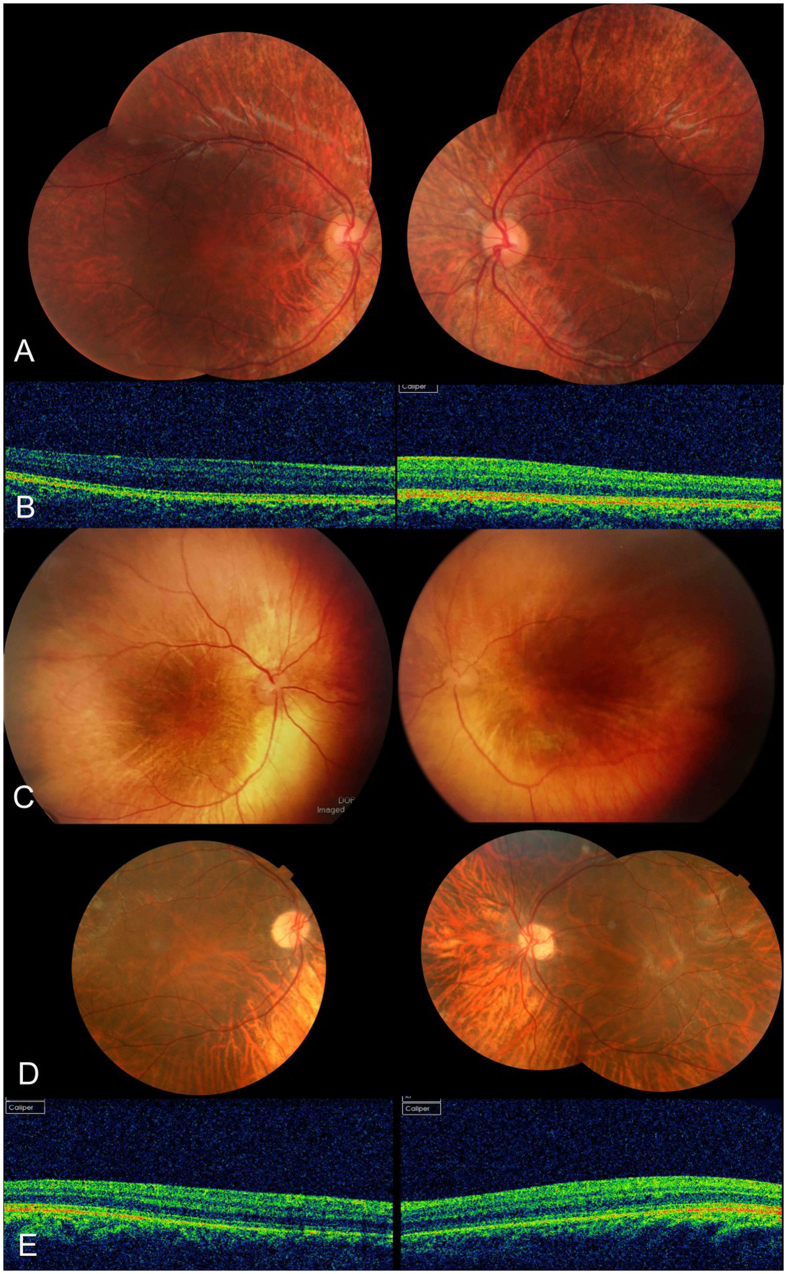
Photographs of fundi from patients. (**A**) Fundus from F1-II:1, (**C**) fundus from F2-II-1, and (**D**) fundus from F3-III:3. (**B**) The OCT of the fovea of F1-II:1, and (**E**) that of F3-III:3. There was hypopigmentation in the posterior of the fundus and severe foveal hypoplasia in the three patients.

**Figure 3:**
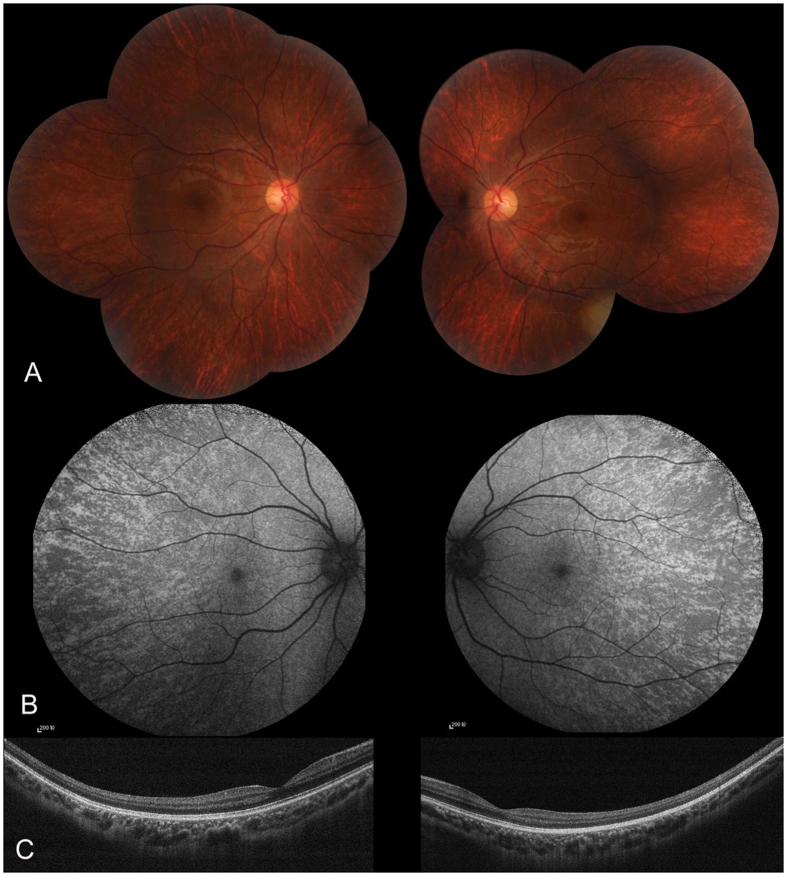
Fundus appearance of the carrier. (**A**) The fundi from carrier F1-I:2 in Family 1. There was pigmentary mosaicism in the retinal pigment epithelium, which is more profound on FAF (**B**). The fovea was normal on OCT, and the retinal structure of the midperipheral area was also normal (**C**).

Three families diagnosed with OA1 were recruited for this study (Fig. 1). The clinical characteristics of OA1 in each affected individual are described in Table 1 and Fig. 2. Table 1 shows the gender, age and clinical characteristics of three patients and one carrier. The fundus images of the three probands, F1-II:1, F2-II:1 and F3-III:3 are shown in Fig. 2. Figure 3 shows the fundus image of a carrier, F1-I:2.

All OA1 patients in this study presented with nystagmus, photophobia and poor visual acuity. Nystagmus was present from birth, and BCVA was between Light Perception (LP) and 0.15. No BCVA data was obtained for Family 1 because the patient would not cooperate with the examination. The VEP and full field ERG were recorded from the proband of Family 3. All ERG waveforms were within normal limits; in particular, the amplitude of scotopic b-wave was close to the upper limit of normal, which is typical for albinism. The VEP showed a decreased amplitude of the P_100_ with normal latency for three channels. They were no differences among the three channels for either eye.

Only the patient from Family 3 had mild iris hypopigmentation. The iris of the other two patients appeared to be normal by slit lamp examination. All three patients exhibited hypopigmentation of the fundus, and all the patients had severe foveal hypoplasia (Fig. 2).

The carrier did not have any symptoms and her BCVA was normal (Table 1). The carrier exhibited normal iris pigmentation; however, the midperiphery of the fundus had alternating streaks that were hypopigmented or normally pigmented, illustrating lyonization, which is more obvious on FAF (Fig. 3). All the participants had normal skin and hair color.

### Mutation analysis

**Figure 4:**
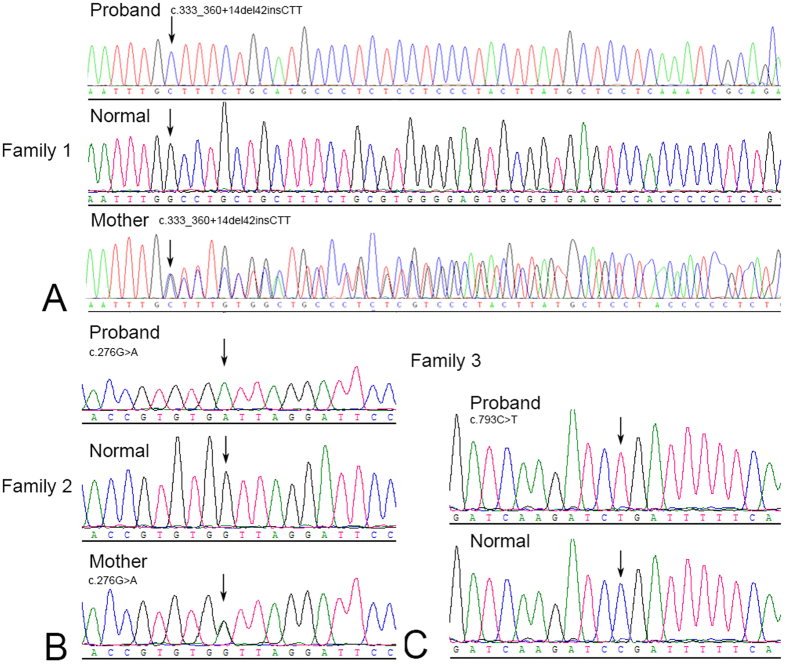
Sequencing analysis of three families with OA1. The direct sequencing image was obtained by direct sequencing. The mutant *GPR143* sequence and corresponding normal sequence are shown for families 1–3 (**A–C**). Each mutation is marked with an arrow.

Sequence analysis of *GPR143* detected three novel mutations in the three families (Table 2, Fig. 4), c.333_360+14del42insCTT, c.276G>A (p.W92X), and c.793C>T (p.R265X), that are predicted to result in loss of function (LOF). These mutations were present in affected males as hemizygous and were heterozygous in female carriers.

## Discussion

In our study, we identified three novel, predicted LOF mutations in *GPR143* among three patients with OA1. All patients presented with severely impaired visual acuity, nystagmus, photophobia, foveal hypoplasia, and fundus hypopigmentation.

Since *GPR143* is the only candidate gene for non-syndromic X-linked ocular albinism, we used Sanger sequencing to confirm the underlying gene mutation. Compared to next generation sequencing (NGS) and whole exome sequencing (WES), Sanger sequencing not only saves on cost but also enhances efficiency when examining a single gene. Among the novel mutations reported in this study, one is a deletion and insertion mutation involving the coding and splicing region and the other two are nonsense mutations. The mutation c.333_360+14del42insCTT involved exon 2 and the splice donor site of second intron of the *GRP143* gene, which may destroy the normal splicing and result in exon skipping or create apremature stop codon. In our study, the two nonsense mutations, p.W92X and p.R265X, would cause a truncated protein. Moreover, the truncated protein is likely to be degraded by nonsense-mediated mRNA decay (NMD), suggesting that both nonsense alleles are LOF. As only three patients were studied, the prevalence of *GPR143* mutations in Chinese patients cannot be conclusively estimated. The *GPR143* mutations reported to date in reported Chinese patients and our data are summarized (Table 2). We found that a deletion/insertion mutation involving the coding region was the most common mutation type (33.3%), followed by nonsense (23.8%) and splice site mutations (23.8%). Missense mutations only accounted for 19.0%. This mutation spectrum is quite different from what has been found in a western population, where approximately 48% of reported OA1 mutations were intragenic deletions, and 43% were missense and nonsense mutations. Bassi and colleagues found a diverse prevalence of large deletions between European (<10%) and North American (>50%) patients with OA1^16^.

The OA1 patients in our study exhibited congenital nystagmus and poor visual acuity with hypopigmentation of the fundus. Iris hypopigmentation ranged from mild to normal. Severe foveal hypoplasia was the prominent clinical feature. The mosaic pattern of the carrier’s fundus is also useful for diagnosis, especially for young children who often are unable to cooperate with a fundus examination. It is unclear how the mutated *GPR143* causes ocular abnormalities. The most prominent signs in affected Caucasians with OA1 are iris translucency, macular hypoplasia, and fundus hypopigmentation^17^,^18^,^19^,^20^. African-American males with OA1 have non-albinotic, moderately pigmented fundi and no translucency of the iris^20^. Japanese patients have fundus hypopigmentation at a level between that of Caucasian and African-American patients^21^,^22^. Therefore, it is postulated that the level of ocular hypopigmentation is related to ethnic origin. In Chinese, iris color is usually brown with little iris translucency, the clinical findings are similar to those in Japanese patients.

The ERG of proband F3-III:3 showed a scotopic b-wave close to the upper limit of normal which conformed to the phenotype. It has been reported that in albinos, amplitudes of the scotopic ERG responses may be greater than normal^23^, which has been postulated to result from the increased amount of stimulating light entering the eye through a hypopigmented anterior segment^24^. In Chinese patients, the pigmentation of the anterior segment is usually normal. We postulate that the relatively elevated scotopic b-wave results from increased scattering of light due to hypopigmentation of fundus. VEP tests have been used to reveal the abnormal optic decussation in albinos^25^. However, we didn’t observe a difference in the amplitudes of the P_100_ for all channels. Both proband F3-III:3 in this study and one OA1 patient in Fang’s study^8^ showed nonspecific VEP findings. Although, chiasmal misrouting is very common in oculocutaneous albinism, it is possible that the misrouting of chiasma is mild in Chinese OA1 patients or minor abnormal chiasmal decussation could not be recorded by VEP.

Iris and fundus hypopigmentation is mild in Chinese OA1 cases^8^. Most cases manifest poor vision at a very young age when it is difficult to perform a detailed clinical examination. OA1 could easily be misdiagnosed as other disease, such as LCA, achromatopsia or CMN. Liu *et al*.^9^ studied a large Chinese family with X-linked recessive inheritance. Without the classical phenotype of OA1, they found that nystagmus was the most prominent and only consistent finding. Patients in the family were initially misdiagnosed as CMN, a congenital abnormality that cause involuntary rhythmic oscillations of the eyes that occurs in the absence of any obvious disorders^26^. The patient from Family 2 was initially misdiagnosed as LCA, since LCA patients also present with poor vision and nystagmus, and clinical examinations are sometimes not available. Genetic testing has helped us establish the exact diagnosis finally. Therefore, the diagnosis of OA1 often requires comprehensive considerations, including retinal structure and function tests, and eventually molecular screening, especially when clinical findings are confusing^18^,^27^.

In summary, this report identified three novel *GPR143* mutations in Chinese patients with OA1. We characterized the clinical manifestations of OA1 in these patients and confirmed that foveal hypoplasia played a vital role in the diagnosis of OA1. Color fundus photos and autofluorescence imaging for female carrier may provide important evidence for diagnosis. Genetic analysis of *GPR143* and detailed clinical examinations are helpful in the early diagnosis and genetic counselling of OA1, avoiding unnecessary and inappropriate intervention.

## Additional Information

**How to cite this article**: Zou, X. *et al*. Molecular genetic and clinical evaluation of three Chinese families with X-linked ocular albinism. *Sci. Rep.* **6**, 33713; doi: 10.1038/srep33713 (2016).

## Figures and Tables

**Table 1:** Summary of clinical findings in affected males and carriers.

ID	Gender	Age	BCVA (right/left)	Iris hypopigmentation	Fundus hypopigmentation	Foveal hypoplasia	Nystagmus
Patient							
F1-II:1	M	4	NA^1^	No	Yes	Yes	Yes
F2-II:1	M	3	LP/LP^2^	No	Yes	Yes	Yes
F3-III:3	M	24	0.15/0.15	Yes	Yes	Yes	Yes
Carrier							
F1-I:2	F	25	1.0/1.0	No	Peripheral mosaic pattern	No	No

^1^Not available, ^2^LP = light perception.

**Table 2:** Summary of *GPR143* mutations in our study and reported Chinese families with OA1.

Author	*GPR143* mutation	Mutation type
Yan, N.^7^	c.807T>A, p.Y269X	Nonsense
Liu, J. Y.^9^	c. 266C>T, p.S89F	Missense
Cai, C. Y.^10^	24422G>C	Splicing
Wang, Y.^12^	c.943G>T, p.G315X	Nonsense
Xiao, X.^11^	Loss of exons 1 and 2	Deletion
Pan, Q.^13^	c.494C>A, p.A165D	Missense
Hu, J.^15^	c.658+1G>T	Splicing
Fang, S.^8^	c.849delT, p.Val284SerfsX15	Deletion
	c.238_240delCTC, p.Leu80del	Deletion
	c.658+1G>A,	Splicing
	c.353G>A, p.G118E	Missense
	g.1103_7266del6164, Loss of exons 2 and 3	Deletion
	g.25985_26546del562, Loss of exon 8	Deletion
Han, R.^14^	c.333G>A, p.W111X	Nonsense
	c.360+1G>C	Splicing
	c.659-1G>A	Splicing
	c.43_50dupGACGCAGC, p.L20PfsX25	Deletion
	c.703G>A, p.E235K	Missense
Our study	c.333_360+14del42insCTT	Deletion and insertion
	c.276G>A, p.W92X	Nonsense
	c.793C>T, p.R265X	Nonsense
